# The effect of *Aspalathin linearis*, *Cyclopia intermedia* and *Sutherlandia frutescene* on sperm functional parameters of healthy male wistar rats

**DOI:** 10.3389/fphys.2023.1211227

**Published:** 2023-06-07

**Authors:** Temidayo S. Omolaoye, Bongekile T. Skosana, Stefan S. du Plessis

**Affiliations:** ^1^ Department of Basic Sciences, College of Medicine, Mohammed Bin Rashid University of Medicine and Health Sciences, Dubai, United Arab Emirates; ^2^ Division of Medical Physiology, Faculty of Medicine and Health Sciences, Stellenbosch University, Tygerberg, South Africa

**Keywords:** sperm, motility, sperm concentration, rooibos (*Aspalathin linearis*), honeybush (*Cyclopia intermedia*), sutherlandia (*Sutherlandia frutescene*)

## Abstract

**Introduction:** Rooibos (*Aspalathin linearis*), honeybush (*Cyclopia intermedia*), and sutherlandia (*Sutherlandia frutescene*) are three Southern Africa indigenous plants, of which the extracts have become house-hold items and are consumed on a large scale. Although, they are known for their antioxidant properties, studies have highlighted danger in the excessive intake. Therefore, the current study investigated whether treatment with rooibos, honeybush, and sutherlandia will impact sperm functional parameters positively or otherwise, in healthy rats.

**Methods:** Fourteen-week-old pathogen-free adult male Wistar rats (250–300 g) were randomly divided into four groups of ten, including a control, rooibos (RF), honeybush (HB) and a sutherlandia (SL) group. After 7 weeks of treatment, animals were sacrificed. Spermatozoa were retrieved from the cauda epididymis for motility, morphology and concentration analysis and the testis was used for all biochemical assays.

**Results:** The infusion treated animals (RF, HB, and SL) presented with a non-significant decrease of −14.3%, −18.2%, −17.2% and −24.8%, −20.7%, −27.3% in total motility and progressive motility when compared to the control group, respectively. There was a significant increase in number of spermatozoa with slow speed (*p* = 0.03), especially in SL treated group compared to the control (*p* = 0.03). Additionally, there was an increase of 28.8%, 31.7%, 23% in superoxide dismutase (SOD) activity of RF, HB and SL compared to control, respectively. This was accompanied with a percentage decrease of −21.1%, −23.7%, 45.9% in malondialdehyde (MDA) levels compared to the control group.

**Conclusion:** In summary, animals treated with the respective infusions presented with a percentage increase in SOD activity but have reduced sperm motility and decreased normal morphology. Paradoxically, they presented with increased sperm concentration. Hence, it is presumed that rooibos, honeybush and sutherlandia may enhance sperm quantity (concentration) but may impair sperm quality (motility morphology) when consumed by healthy animals.

## 1 Introduction

The use of medicinal plants in treating diseases have been used over many decades in traditional medicine. It is interesting to know that the world is becoming more intrigued in exploring the benefits of phytochemicals and to also investigate their importance in health.

Rooibos, honeybush and sutherlandia are indigenous Southern Africa plants, and they are caffeine-free beverages. Rooibos originate from the Cederberg Mountains of the Western Cape region of South Africa (McGaw et al., 2007; Joubert et al., 2008). The leaves and stems are used for the commercially available rooibos tea. Rooibos is generally available either as the unfermented (green rooibos) or the fermented (reddish-brown rooibos) form. The fermented tea is achieved by oxidation that result in the unique reddish-brown leaf colour with woody-fynbos-floral honey flavour (Koch et al., 2012). Whether unfermented or fermented rooibos, studies have shown the presence of numerous bioactive chemical compounds, which include nothofagin, aspalathin, C-5-hexosyl derivative of aspalathin, aspalalinin, phenypyruvic acid-2-0-glucoside, orientin, isoorientin, vitexin, isovitexin, luteolin, luteolin-7-0-glucoside, chyrysoerio, and many more (Beelders et al., 2012). Due to the presence of high aspalathin levels and other phytochemical contents, hot water extracts prepared from green and fermented rooibos are used as food and/or cosmetic ingredients. Although the aspalathin content of unfermented rooibos is greater (8%) than the fermented (2%), the latter is still more preferable in food because of the flavour, and it is considered more economical as there is a greater demand for it (Joubert and de Beer, 2012; de Beer et al., 2017). Studies have elucidated the role of rooibos in protecting against disease development (Lawal et al., 2019) and also the ameliorative effects on diverse disorders (Marnewick et al., 2005; Ajuwon et al., 2018). Akinrinmade et al. suggested that the long-term continuous consumption of fermented rooibos tea confer a mild neuroprotection against ischaemic brain injury in rats. This was evidenced by the decrease in brain oedema, neuronal apoptosis, decreased lipid peroxidation and increase total antioxidant capacity, which cumulatively improved neuro-behavioural outcomes in rats (Akinrinmade et al., 2017). Nash et al. reported that osteoblast (Saos2 cells) treated with luteolin and orientin (from rooibos) displayed elevated alkaline phosphatase and mitochondrial activities. This was associated with reduced toxicity and decreased expression of anti-inflammatory cytokines, which collectively suggest that these rooibos metabolites improved osteoblast mineral content (Nash et al., 2015). Other studies have reported its antioxidant (Hong et al., 2014), anti-diabetic (Son et al., 2013; Dludla et al., 2018; Layman et al., 2019; Orlando et al., 2019) and anti-inflammatory (Pyrzanowska et al., 2019) properties.

Honeybush is native to the Southeast and Southwest coastal areas of South Africa, and it forms a part of the fynbos biome with the family name Fabaceae. It has been shown to be used as a traditional tea since the 19th century (Joubert et al., 2011; Schloms and Swart, 2014). Honeybush tea does not only have a pleasant taste and scent, it also contains phytochemicals and volatile organic compounds that are beneficial to health (Joubert et al., 2008). The known health benefits of honeybush are ascribed to its rich bioactive compounds such as Eriodictyol, Hesperetin, Isokuranetin, Naringenin, Chrysoeriol, Luteolin, Kaempferol, Afrormosin, Calycosin, Formononetin, Fujikinetin, Pseudobaptigen, Wistin, Flemichapparin, Medicagol, Sophoracoumestan, Pinitol, Isomangiferin and Mangiferin (Kamara et al., 2003; Jang et al., 2008).

Choi et al. reported that honeybush improved skin wrinkles, elasticity and hydration in patients with crow’s feet wrinkles after daily supplementation during their randomized double-blinded controlled trial (Choi et al., 2018). The anti-wrinkle effect of honeybush has been reported in animals (Im et al., 2014) and *in vitro* studies (Magcwebeba et al., 2016). Furthermore, studies have also shown its antioxidant (Sánchez et al., 2000; Marnewick et al., 2003), anti-mutagenic (Kokotkiewicz and Luczkiewicz, 2009) and mucosa immune therapy (Murakami et al., 2018) effects in both *in vivo* and *in vitro* studies.

Sutherlandia has been used in traditional medicine since the 1800s. Sutherlandia has a variety of health benefits which are associated to its bioactive phytochemicals. The chemical compounds include, asparagine, proline and arginine (Moshe et al., 1998; van Wyk and Albrecht, 2008), ƴ-aminobutyric acid (GABA), L-carvanine, (Ortega, 2003), sutherlandin A, sutherlandin B, sutherlandin C, and sutherlandin D) (Avula et al., 2010), sutherlandioside A, sutherlandioside B, sutherlandioside C, sutherlandioside D (Avula et al., 2010) and cycloartane-type triterpene glycoside (van Wyk and Albrecht, 2008). Based on the array of pharmacological products present in sutherlandia, studies have highlighted its role in cancer (Tai et al., 2004; Grandi et al., 2005), Human immunodeficiency virus/acquired immunodeficiency syndrome (HIV/AIDS) (Harnett et al., 2005; Mills et al., 2005; Wilson et al., 2015), diabetes (Sia, 2004; Chadwick et al., 2007; Mackenzie. et al., 2012), inflammation (Lei et al., 2015; Vasaikar et al., 2018) stress (Chuang et al., 2015; Sergeant et al., 2017) and oxidative stress (Fernandes et al., 2004; Tobwala et al., 2014).

With regards to the effects of these infusions on male reproduction, very few studies are available. This include a study that reported improvement in sperm motility, sperm concentration and sperm viability after treatment with unfermented rooibos in rats (Opuwari and Monsees, 2014). These authors investigated the effects of unfermented and fermented rooibos teas on male reproductive parameters when both forms are administered to rats at varying concentrations (2% or 5%) for 52 days. Their findings showed that although the sperm parameters of animals treated with unfermented rooibos significantly improved, that of those in the fermented groups either showed non-significant increasing trend or no change (Opuwari and Monsees, 2014). Ros-Santaella and Pintus showed that the *in vitro* treatment of boar semen with rooibos protected the sperm acrosome structure, which was accompanied by improved sperm velocity (Ros-Santaella and Pintus, 2017). Additionally, Awoniyi et al. suggested that rooibos may provide a measure of protection against induced oxidative damage by elevating antioxidant activities and thus improve sperm function (Awoniyi et al., 2012).

However, to the best of our knowledge, there are no studies reporting the effect of honeybush or sutherlandia on male reproduction. Despite the numerous health benefits of these infusions, there are concerns as to their safety in long-term use (Fantoukh et al., 2019). Opuwari and Monsees reported that lengthened exposure to rooibos induced acrosome reaction in rats, which can subsequently lead to impaired reproduction (Opuwari and Monsees, 2014). Additionally, the toxicity of canavanine (from sutherlandia) has been implicated in lupus erythematous syndrome. Long-term exposure to sutherlandia have likewise been reported to cause protein cross-links and it can as well result in autoimmunity and teratogenicity. Ngcobo et al. showed that the *in vitro* chronic treatment of normal T-lymphocyte cells with sutherlandia was toxic (Ngcobo et al., 2012), which means too much of sutherlandia may be hazardous. Therefore, the current study was designed to investigate whether treatment with rooibos, honeybush and sutherlandia will impact sperm functional parameters positively or otherwise, in healthy rats.

## 2 Materials and methods

### 2.1 Animal care

Fourteen-week-old healthy pathogen-free adult male Wistar rats (250–300 g) were housed at the Stellenbosch University’s Faculty of Medicine and Health Sciences’ Animal Unit at room temperature (18°C–23°C), under a normal 12:12 light/dark cycle. Animals were caged individually, had free access to food (standard raw chow) and water/infusions and were treated according to the recommendations of the Laboratory Animal Care of the National Society of Medical Research and the National Institutes of Health Guide for the Care and Use of Laboratory Animals (Maget, 1953). Ethics approval was obtained from the Stellenbosch University Animal Ethics Committee (SU-ACUD17-00016).

### 2.2 Infusion preparation

Fermented rooibos (2%), fermented honeybush (4%) and unfermented sutherlandia (0.2%) were prepared according to standard protocols. These concentrations were based on previously published studies. Preparation procedure conformed to the experimental established protocols for rooibos (Marnewick et al., 2011), honeybush (Du Toit and Joubert, 1999) and sutherlandia (Tobwala et al., 2014). In brief, 2% rooibos was prepared by adding 20 g of dried fermented rooibos in 1 L of boiling water and allowed to steep/rest for 30–45 min. The mixture was filtered three times using a cheesecloth, number 4 filter paper and number 1 filter paper (Whatman™, Buckinghamshire, United Kingdom) respectively. Filtered infusions were transferred to a dark bottle plastic container and stored at 4°C. Honeybush (4%; 40 g in 1 L) was prepared following the similar steps as used for making rooibos tea. However, due to the bitter taste of sutherlandia, the concentration was reduced to 0.2%. Briefly, 40 g of unfermented sutherlandia was prepared as described for rooibos, from the initial 4% solution, 2.5 mL was diluted in 50 mL of water to give the working concentration of 0.2%.

### 2.3 Experimental design

Forty animals were randomly divided into four groups of ten. The groups include a control group, which received tap water, rooibos group (RF) (2% fermented), honeybush (HB) (4% fermented) and a sutherlandia group (SL) (0.2% unfermented). Animals had access to food and fluid *ad libitum*. In the case of the treatment groups, the animals received the treatment through different and corresponding fluid infusions. Hence, as water was the only drinking source for the control animals, the respective infusions were the only drinking source for the animals in the treatment groups. During the first week of acclimatization, animals were fed with pellets (standard Epol™ rat chow) and water only. However, after the acclimatization period, rats were fed with standard rat chow, water, and the different infusions as appropriate. To keep up with the monitoring of the animals, food and fluid intake of the animals were measured three times weekly.

Animals were sacrificed after 7 weeks of treatment. Blood, testis, and epididymis were harvested. Blood was collected into a heparized tube, placed on ice for 30 min and afterwards centrifuged (3000 rpm at 4°C for 30 min). Plasma was stored at −80°C until further analysis. The testis was used for all biochemical analysis, while the caudal area of the epididymis was dissected for sperm retrieval. Relative testicular/epididymal weight were calculated by expressing the tissue weight as a percentage of body weight.

### 2.4 Sperm functional parameters

#### 2.4.1 Motility

Sperm retrieval and motility analysis were carried out as described by (Omolaoye et al., 2018). Briefly, the left epididymis was rinsed in a Petri dish containing 2 mL Hams F-12 nutrient media (Sigma Chemicals, St Louis, MO, United States) at 37°C. The caudal area of the rinsed epididymis was dissected, and spermatozoa were retrieved into a separate 2 mL Hams F-12, at 37°C. After 30 s of retrieval, 2 µL of the sperm solution was infused into a 20 µL depth chamber slide (Leja, Netherlands) and placed on a Nikon Eclipse E200 microscope with an in-built heating stage (37°C). Sperm motility was measured via computer-aided sperm analysis (CASA) using the Sperm Class Analyser (SCA 6.3, Microptic, Barcelona, Spain). Total motility, progressive motility and the sperm kinematic parameters (curvilinear velocity (VCL), average path velocity (VAP), straight-line velocity (VSL), straight-line index (STR), linearity index (LIN), sperm oscillation index (WOB), amplitude lateral head (ALH) and beat frequency (BCF) were analysed. After analysing sperm motility at 30 s, sperm concentration was measured by dissecting the caudal epididymis into smaller pieces and was left for a further 5 min allowing a maximum number of spermatozoa to swim out. The pieces were removed after 5 min, and the sperm solution was mixed homogenously. Of the 2 mL solution, 10 µL was diluted in 50 µL of Hams F-12 and sperm concentration was analysed through CASA.

#### 2.4.2 Morphology

From the sperm solution used for sperm concentration analysis, 10 µL was smeared on a glass slide and allowed to air dry. The dried slides were stained with Sperm Blue^®^ fixative and stain (Microptic SL, Barcelona, Spain) for 3 min, rinsed for 3 s to remove excessive stain, air dried and then mounted (DPX, HiMedia Laboratories Pvt. Ltd., Mumbai, India). Sperm morphology was analysed through computer-aided sperm morphometry analysis (CASMA) using the SCA^®^ (Maree et al., 2010).

### 2.5 Testosterone and estradiol

The plasma concentration of testosterone (E-EL-0072) and estradiol (E-EL-0065) were measured using a commercially available ELISA kit (Elabscience Biotechnology, Hubei) as per manufacturer’s instructions.

### 2.6 Biochemical analysis

#### 2.6.1 Superoxide dismutase (SOD) activity

Testicular tissue samples were homogenized in lysis buffer (Na_3_PO_4_, 0.5% Triton X-100) and centrifuged at 15000 rpm for 20 min at 4°C. Tissue homogenates were diluted to 10x in deionized water. From the diluted standards and samples, 10 μL were dispensed into the microplate wells in triplicate, followed by adding 170 μL of diethylenetriaminepentaacetic acid (DETAPAC) and 5 µL of SOD assay buffer (50 mM Na/K Phosphate buffer at pH 7.4). SOD activity was measured on a plate reader (Multiskan spectrum) at 490 nm, 25°C for 5 min at 1 min interval using SkanIt RE for MSS 2.2 (ThermoScientific™ Inc. immediately after adding 15 μL of freshly prepared 6-hydroxydopamine (6-OHD) into the well.

#### 2.6.2 Catalase

Tissue homogenates were obtained as described for SOD. From the diluted samples and standards, 5 μL were loaded in triplicate into UV microplate wells. Catalase assay buffer (170 μL) were added into each well and lastly, 50 μL of H_2_O_2_ was added into the wells and analysis was performed immediately on a plate reader (Multiskan spectrum) at 240 nm every 60 s over a 5 min period using SkanIt RE for MSS 2.2 (ThermoScientific™ Inc.) software.

#### 2.6.3 Malonaldehyde (MDA) levels

Testicular tissue samples were homogenized in lysis buffer (0.1 M KPi, 1.15% KCl) by bullet blending at speed 9 for 3 min with a 1-min interval in-between. MDA levels in the testis was determined by pipetting 100 µL of standards and samples into corresponding 10 mL glass tubes, followed by adding 1 mL of SDS and 2 mL of 10%TCA-BHT buffer solution. Samples were vortexed, and after resting for 10 min, 2 mL of TBA was added and vortexed again. The standards and samples were covered with marbles and incubated in a water bath (1 h at 100°C), where after it was cooled on ice for 15 min. The standards and samples were centrifuged (3000 rpm, 15 min 4°C) and the supernatants retrieved. From the supernatants, 250 μL of each standard and sample were loaded in triplicate into microplate wells and analysed on a plate reader (Multiskan spectrum) at a 532 nm wavelength within 30 min after centrifugation.

### 2.7 Statistics

GraphPad Prism™ software (GraphPad™ Software, Version 8.2, CA, United States) was used. Normal data distribution was measured using the Anderson-Darling and Kolmogorov-Smirnov normality tests. When data passed all normality tests, a one-way ANOVA of variance with a Tukey’s Post-hoc Test were performed. Where data were not evenly distributed, a Kruskal–Wallis test and a Dunns Post-hoc Test were carried out. A probability level of *p* < 0.05 was considered statistically significant and results are expressed as mean ± SD.

## 3 Results

### 3.1 Anthropometric data

After 7 weeks of treatment, no significant differences were observed in the body, testicular and epididymal weights between any of the groups (Table 1). However, the SL group presented with a higher (7%) percentage epididymal weight mean compared to the RF group (*p* = 0.07). The RF treated animals displayed a significantly lower (−7.5%) relative testicular weight compared to the HB group (*p* = 0.04), while there was no significant difference in relative epididymal weight between the groups (*p* = 0.3) (Table 1).

**TABLE 1:** Body and tissue weights.

Parameters	Control	Rooibos	Honeybush	Sutherlandia	Global *P*
Bodyweight (g)	344,6 ± 19,04	349,4 ± 21,41 (1.4%)	345,3 ± 25,55 (0.2%)	352,6 ± 19,37 (2.3%)	0,8224
Testicular weight (g)	1,414 ± 0,12	1,346 ± 0,08 (−4.8%)	1,438 ± 0,12 (1.7%)	1,423 ± 0,11 (0.6%)	0,1492
Epididymal weight (g)	0,4702 ± 0,03	0,4560 ± 0,025 (−3.0%)	0,4753 ± 0,046 (1.1%)	0,4892 ± 0,022 (4.0%)	0,0618
Relative testicular weight (%)	0,4107 ± 0,03	0,3858 ± 0,025^a^ (−6.1%)	0,4174 ± 0,034 (1.6%)	0,4034 ± 0,018 (−1.8%)	0,0588
Relative epididymal weight (%)	0,1367 ± 0,01	0,1308 ± 0,008 (−4.3%)	0,1381 ± 0,015 (1.0%)	0,1389 ± 0,004 (0.6%)	0,3406

^a^ *p* < 0.05 vs. Honeybush, values in brackets () denotes the percentage change from control, *n* = 10.

### 3.2 Sperm functional parameters

**FIGURE 1:**
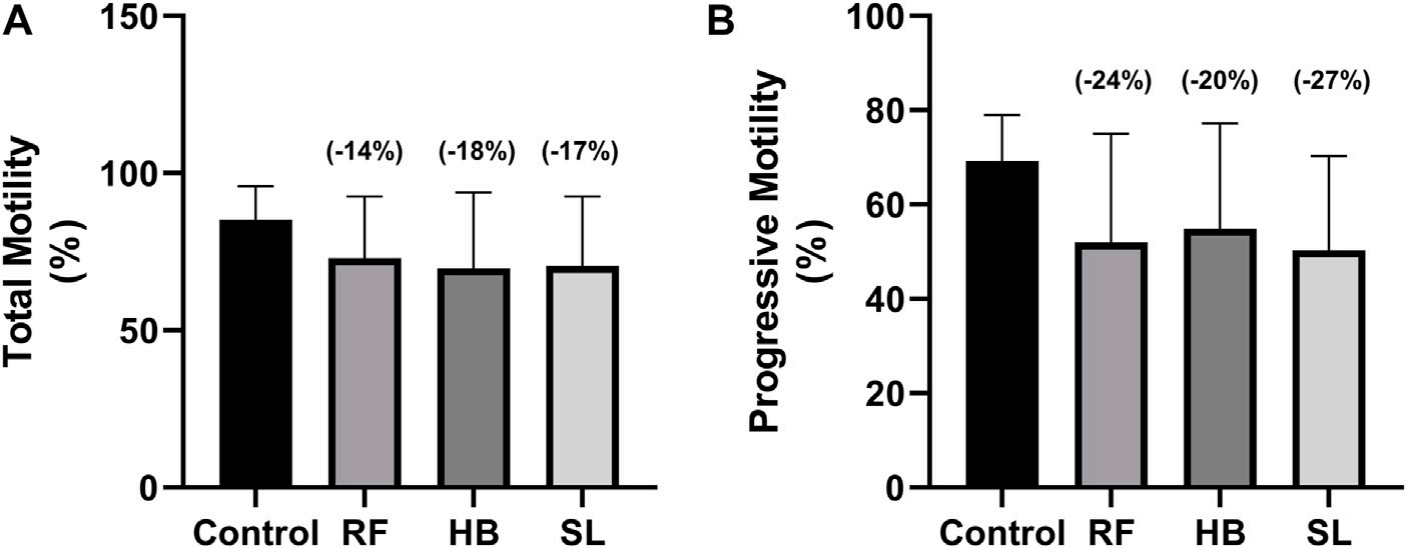
Sperm total and progressive motilities at 30 s. **(A)** total motility, **(B)** progressive motility, RF = rooibos, HB = honeybush, SL = sutherlandia, values in brackets () denotes the percentage change from control, *n* = 10.

**FIGURE 2:**
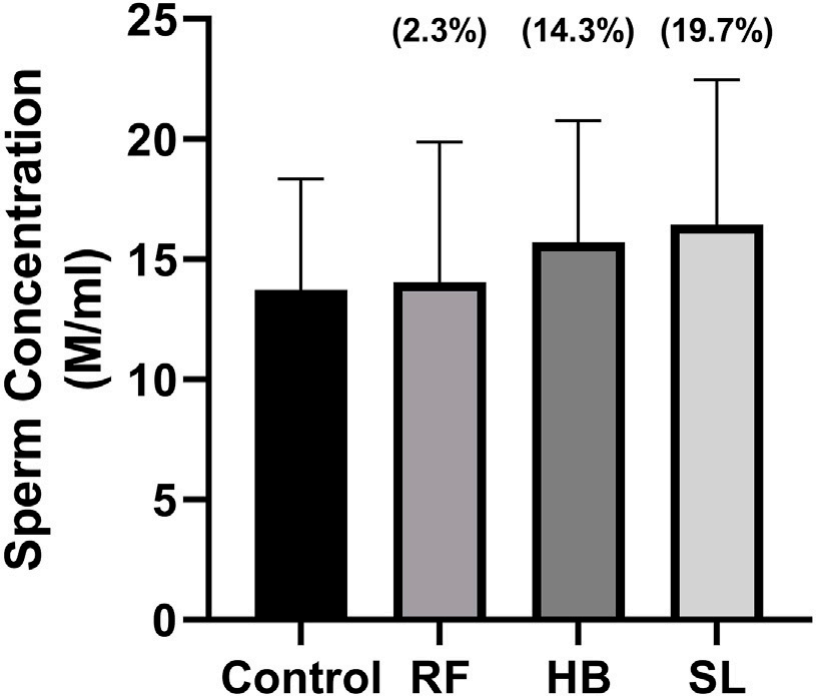
Sperm concentration measured after 5 min of retrieval. RF = rooibos, HB = honeybush, SL = sutherlandia, values in brackets () denotes the percentage change from control, *n* = 10.

The infusion treated animals (RF, HB, and SL) presented with a non-significant decrease (−14.3%, −18.2%, −17.2%) (−24.8%, −20.7%, −27.3%) in total motility and progressive motility when compared to the control group respectively (Figures 1A, B). Although not significant, the rapid progressive motility of RF, HB and SL animals reduced by 23%, 28% and 37% respectively (Table 2), and there was a significant increase in the number of spermatozoa with slow speed (*p* = 0.03), especially the SL treated group compared to the control (*p* = 0.03) (Table 2). All sperm kinematic parameters of the infusion treated animals were non-significantly reduced compared to the control (Table 3). RF, HB and SL treated animals displayed a decrease in VCL (−14.3%, −18.8%, −23.7%); VAP (−16.5%, −22%, −27.6%); VSL (−20%, −25.5%, −33.2%); STR (−11.1%, −8.5%, −14.9%); LIN (−15.8%, −14.6%, −22.6%) WOB (−8%, −9%, −10%); ALH (−14.8%, −15.9%, −20.5%) and BCF (−16.3%, −18.7%, −26.3%), compared to the control group respectively (Table 3). Interestingly, infusion treated groups displayed a non-significant increase in sperm concentration compared to the control group (Figure 2). However, there was no significant differences in the percentage of morphologically normal spermatozoa between the groups, but RF and SL are on the decrease compared to the control (Table 4). Taken together, findings showed that the percentage of rapidly progressive motile spermatozoa follows the order of control > RF > HB > SL.

**TABLE 2:** Sperm speed and progressive motilities as measured at 30 s

Parameters	Control	Rooibos	Honeybush	Sutherlandia	Global *P*
Rapid speed (%)	53,71 ± 18,53	42,88 ± 24,49 (−20.2%)	38,68 ± 24,10 (−31.7%)	36,94 ± 22,33 (−31.2%)	0,3534
Medium speed (%)	17,64 ± 9,681	9,319 ± 6,486 (−47.2%)	12,43 ± 6,924 (−29.5%)	10,24 ± 6,747 (−41.9%)	0,0820
Slow speed (%)	13,79 ± 5,225	20,68 ± 8,814 (49.9%)	18,52 ± 5,651 (34.3%)	23,27 ± 8,272^a^ (68.7%)	0,0369
Rapid progressive motility (%)	24,19 ± 11,35	18,61 ± 12,91 (23%)	17,38 ± 12,00 (28%)	15,08 ± 9,40 (37%)	0,2919
Medium progressive motility (%)	41,59 ± 10,73	29,83 ± 22,41 (−28.2%)	29,77 ± 17,24 (−28.4%)	27,15 ± 16,92 (−34.7%)	0,2619
Non-progressive motility (%)	19,36 ± 6,893	24,45 ± 8,454 (26.2%)	22,48 ± 6,192 (16.1%)	28,23 ± 7,948 (45.8%)	0,0746

^a^ *p* < 0.05 vs. control, values in brackets () denotes the percentage change from control, *n* = 10.

**TABLE 3:** Sperm kinematic parameters as measured at 30 s of retrieval.

Parameters	Control	Rooibos	Honeybush	Sutherlandia	Global *P*
VCL (µm/s)	191,1 ± 48,70	163,7 ± 40,97	155,1 ± 35,23	145,8 ± 30,83	0,0799
VAP (µm/s)	87,85 ± 25,67	73,29 ± 28,39	68,47 ± 20,24	63,52 ± 21,67	0,2031
VSL (µm/s)	53,55 ± 18,42	42,75 ± 15,01	39,87 ± 12,19	35,77 ± 13,33	0,1217
STR (%)	58,55 ± 5,960	52,03 ± 6,885	53,56 ± 7,608	49,80 ± 7,788	0,0577
LIN (%)	28,53 ± 3,798	24,00 ± 7,050	24,36 ± 6,019	22,08 ± 6,994	0,1348
WOB (%)	45,99 ± 3,302	42,29 ± 9,588	41,84 ± 6,011	41,25 ± 8,967	0,4825
ALH (µm)	7,360 ± 1,363	6,269 ± 1,688	6,186 ± 1,451	5,846 ± 1,063	0,1088
BCF (Hz)	16,65 ± 3,116	13,92 ± 3,311	13,53 ± 2,933	12,26 ± 4,292^a^	0,0504

^a^ *p* < 0.05 vs. control, *n* = 10.

**TABLE 4:** Sperm normal morphology and morphometric parameters.

Parameters	Control	Rooibos	Honeybush	Sutherlandia	Global *P*
Head arc	21,70 ± 0,491	21,47 ± 0,770 (−1.0%)	21,59 ± 0,440 (−0.5%)	21,96 ± 0,319 (1.2%)	0,2159
Linearity	53,10 ± 2,126	52,43 ± 2,857 (1.3%)	52,35 ± 1,550 (−1.4%)	52,50 ± 1,984 (−1.1%)	0,8636
Width	1,436 ± 0,103	1,467 ± 0,083 (2.15%)	1,451 ± 0,117 (1.0%)	1,409 ± 0,148 (−1.88%)	0,5116
Normal morphology (%)	59,60 ± 11,350	58,70 ± 9,262 (−1.5%)	59,80 ± 16,210 (0.3%)	54,20 ± 17,780 (−8.3%)	0,7907

Values in brackets () denotes the percentage change from control, *n* = 10.

### 3.3 Hormones and biochemical assays

**FIGURE 3:**
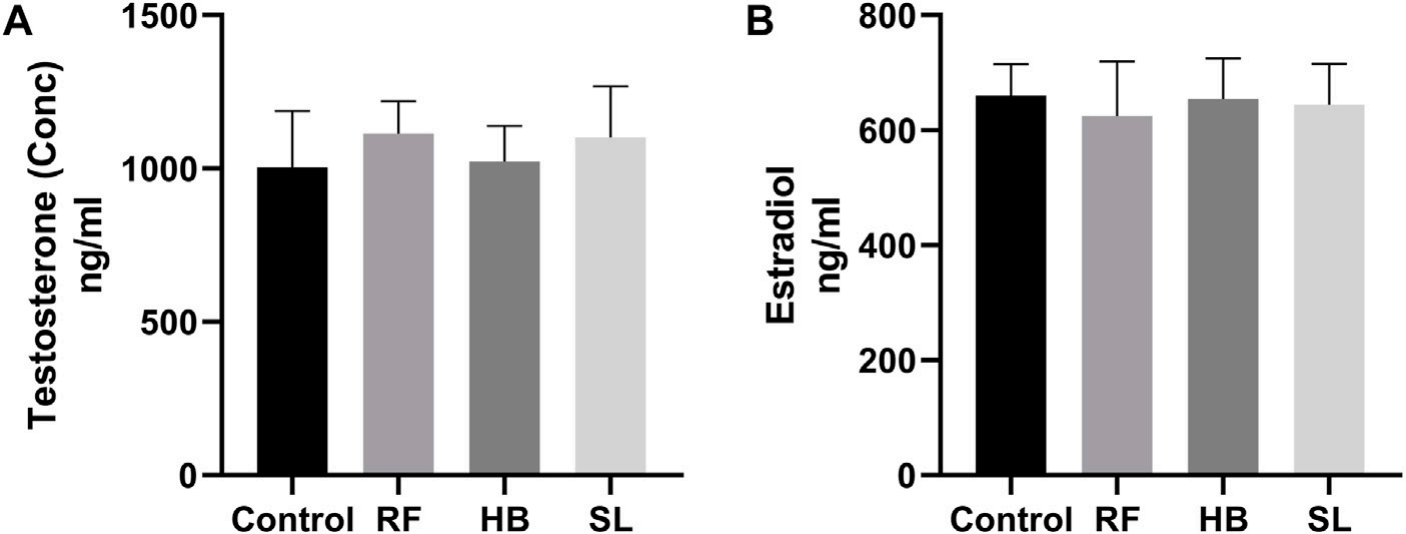
Testosterone and estradiol concentration. **(A)** testosterone, **(B)** estradiol, RF = rooibos, HB = honeybush, SL = sutherlandia, *n* = 9.

**FIGURE 4:**
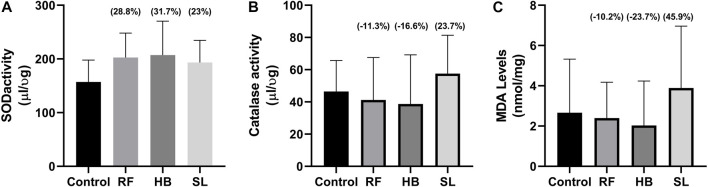
Oxidative stress parameters. **(A)** SOD activity, **(B)** catalase activity, **(C)** MDA levels, RF = rooibos, HB = honeybush, SL = sutherlandia, values in brackets () denotes the percentage change from control, *n* = 10.

There were no statistical difference in the concentration of testosterone and estradiol between the groups (Figures 3A, B). However, there was an increase of 28.8%, 31.7%, and 23% in SOD activity, decrease of −11.3%, −16.6%, 23.7% in catalase activity and a decrease of −21.1%, −23.7%, 45.9% in MDA levels of RF, HB and SL groups when compared to the control group respectively (Figures 4A–C).

## 4 Discussion

The use of natural products in the prevention and treatment of diverse diseases is increasing. This is due to the availability of phytochemical bioactive compounds in these plants, which are able to mimic the pharmacokinetics of synthetic drugs with fewer resultant adverse effects (Mills et al., 2005; Jang et al., 2008; van Wyk and Albrecht, 2008). Rooibos, honeybush and sutherladia are plants with bioactive compounds and they have been shown to have numerous health benefits (van Wyk and Albrecht, 2008). However, only a few studies have been performed regarding their effects on male reproductive function. From these few studies, concerns regarding long term use have already been highlighted. Opuwari and Monsees reported that long-term consumption of rooibos resulted in spontaneous acrosome reaction, which may impair male reproduction (Opuwari and Monsees, 2014). The current study investigated the effects of rooibos, honeybush and sutherlandia on male reproductive functional parameters in rats.

After 7 weeks of treatment, there was no significant difference in body and organ (testes and epididymides) weights. This is similar to the report of Opuwari and Monsees, who also showed no significant difference in the body and tissue weight gain after 52 days of rooibos consumption in rat (Opuwari and Monsees, 2014). Many natural products of plant origin contains flavonoids, and flavonoids are considered endocrine disruptors (Patisaul and Jefferson, 2010). Chandra et al. reported that green tea administered in relatively high dose inhibited the activities of testicular 3β and 17β-hydoxy-steriod dehydrogenase, decreased serum testosterone and reduced testicular weight, which cumulatively resulted in inhibition of spermatogenesis. This suggest that high dose of this tea may impair the morphological and normal function of the testis (Das and Karmakar, 2015). Regarding rooibos, an *in vitro* study reported decrease in the production of testosterone by the Leydig cells after treatment with rooibos (Opuwari and Monsees, 2015). However, In the current, although RF animals presented with a decrease in relative testicular weight, there was no significant difference in the serum concentration of testosterone, but the estradiol levels decreased by 5%. The observed result of testosterone in the current study is supported by a study that reported no significant difference in serum testosterone after administration of both fermented and unfermented forms of rooibos in rats (Opuwari and Monsees, 2014). The role of rooibos as endocrine disruptor still remains inconclusive, because, recently, Noh reported that MR-10, a novel complex of dandelion and rooibos increases testosterone levels and also improved sperm production in older (>45) men (Noh, 2018). Hence, more studies are required to ascertain the role of rooibos on the endocrine regulation of male reproduction. Honeybush has been shown to have mild oestrogenic activity as it was reported to induce proliferation of oestrogen-insentive MDA-MD-231 cells (Verhoog et al., 2007). As previously stated, studies are lacking on its role in male reproduction, thus more studies are required to investigate the role on male reproduction.

In the current study, the effects of rooibos, honeybush and sutherlandia on the sperm motility, concentration and morphology represent an ilicit/confusing situation. Animals treated with the respective infusion (RF, HB, SL) presented with a decrease in all sperm kinematic parameters. This is in contrast to the report of Ros-Santaella and Pintus. They showed that after treating boar semen with rooibos, there was an increase in sperm kinematic parameters (Ros-Santaella and Pintus, 2017). The contrasting result may be due to the difference in models. Additionally, there was a decrease in the total motility (−14%, −18%, −17%), progressive motility (−24%, −20%, −27%) and normal sperm morphology (−1.5%, 0.3% and −9%) of animals treated with RF, HB and SL, with SL animals showing a significant increase in the percentage of spermatozoa with slow speed. The results of the total and progressive motilities of the current study are in contrast with the reports of Awoniyi et al. and Ayeleso et al. (Awoniyi et al., 2012; Ayeleso et al., 2014b). The difference may be due to the health status of animals analysed. Awoniyi et al. reported an increase in the total and progressive motilities of diabetic animals treated with rooibos and Ayelso et al. showed an improvement in the total and progressive motilities of animals that were induced with OS. Additionally, from data obtained in our laboratory (SURRG Stellenbosch University Reproductive Research Group), we observed that diabetic animals treated with fermented rooibos displayed an increase that was up to 5% in total and progressive motilities when compared to non-diabetic animals (Omolaoye et al., 2021). This suggest that rooibos consumption may be beneficial in a diseased state, especially, DM-related male reproductive function impairment in rats. However, when consumed by healthy rats, it may reduce sperm motility. Since studies on honeybush and sutherlandia are lacking, little can be said about them. We can speculate that when administered to healthy animals, there is a possibility of sperm function impairment since they follow the same trend as rooibos. Nevertheless, if the three infusions are compared, findings showed that the percentage of rapidly progressive motile spermatozoa follows the order of control > RF > HB > SL.

Interestingly, animals treated with these infusions displayed a non-significant increase in sperm concentration. This is partly supported by Opuwari and Monsees. They reported that unfermented rooibos supplementation significantly enhanced sperm concentration in rats (Opuwari and Monsees, 2014). Several studies have shown the antioxidant potential of rooibos, honeybush and sutherlandia on diverse diseases (Fernandes et al., 2004; Ayeleso et al., 2014a). In like manner, the RF, HB and SL animals of the current study presented with an increase in SOD activity accompanied with reduced MDA levels in RF and HB groups, with SL group showing higher MDA levels. The former is supported by several studies that have shown increased testicular antioxidant enzyme activity after treatment with rooibos either in disease or health (Awoniyi et al., 2012; Ayeleso et al., 2014b; Opuwari and Monsees, 2014). Although, SOD activity was increased in the infusion treated groups of the current study, the sperm motility and morphology were adversely affected. The mechanisms behind these outcomes were not investigated, but it can be speculated that, the increased SOD and reduced catalase activities observed are compensatory responses. As SL that displayed higher SOD and catalase activities, resultantly showed a non-significant 45% increase in MDA levels.

## 5 Conclusion

The present study evaluated the role of rooibos, honeybush and sutherlandia on sperm functional parameters in healthy rats. Animals treated with the respective infusions presented with percentage increase in antioxidant enzyme activity but have reduced sperm motility and decreased normal morphology. Paradoxically, they presented with increased sperm concentration. Hence, it is concluded that rooibos, honeybush and sutherlandia may enhance sperm concentration, which represent sperm quantity, but they may impair sperm motility and morphology (sperm quality) when consumed by healthy animals. Thus, caution should be taken regarding the quantity of teas consumed when healthy, as it can affect the antioxidant shift (antioxidant paradox). That is, too much of antioxidants can push into the opposite state of oxidative stress and rather lead to reductive stress.

## Data Availability

The original contributions presented in the study are included in the article/supplementary material, further inquiries can be directed to the corresponding author.
